# Multiomic Integration of Public Oncology Databases in Bioconductor

**DOI:** 10.1200/CCI.19.00119

**Published:** 2020-10-29

**Authors:** Marcel Ramos, Ludwig Geistlinger, Sehyun Oh, Lucas Schiffer, Rimsha Azhar, Hanish Kodali, Ino de Bruijn, Jianjiong Gao, Vincent J. Carey, Martin Morgan, Levi Waldron

**Affiliations:** ^1^ Graduate School of Public Health and Health Policy, City University of New York, New York, NY; ^2^ Institute for Implementation Science and Population Health, City University of New York, New York, NY; ^3^ Roswell Park Comprehensive Cancer Center, Buffalo, NY; ^4^ Section of Computational Biomedicine, Boston University School of Medicine, Boston, MA; ^5^ Department of Healthcare Policy and Research, Weill Cornell Medicine, New York, NY; ^6^ Marie-Josée and Henry R. Kravis Center for Molecular Oncology, Memorial Sloan Kettering Cancer Center, New York, NY; ^7^ Department of Epidemiology and Biostatistics, Memorial Sloan Kettering Cancer Center, New York, NY; ^8^ Channing Division of Network Medicine, Brigham and Women’s Hospital, Harvard Medical School, Boston, MA

## Abstract

**PURPOSE:**

Investigations of the molecular basis for the development, progression, and treatment of cancer increasingly use complementary genomic assays to gather multiomic data, but management and analysis of such data remain complex. The cBioPortal for cancer genomics currently provides multiomic data from > 260 public studies, including The Cancer Genome Atlas (TCGA) data sets, but integration of different data types remains challenging and error prone for computational methods and tools using these resources. Recent advances in data infrastructure within the Bioconductor project enable a novel and powerful approach to creating fully integrated representations of these multiomic, pan-cancer databases.

**METHODS:**

We provide a set of R/Bioconductor packages for working with TCGA legacy data and cBioPortal data, with special considerations for loading time; efficient representations in and out of memory; analysis platform; and an integrative framework, such as MultiAssayExperiment. Large methylation data sets are provided through out-of-memory data representation to provide responsive loading times and analysis capabilities on machines with limited memory.

**RESULTS:**

We developed the curatedTCGAData and cBioPortalData R/Bioconductor packages to provide integrated multiomic data sets from the TCGA legacy database and the cBioPortal web application programming interface using the MultiAssayExperiment data structure. This suite of tools provides coordination of diverse experimental assays with clinicopathological data with minimal data management burden, as demonstrated through several greatly simplified multiomic and pan-cancer analyses.

**CONCLUSION:**

These integrated representations enable analysts and tool developers to apply general statistical and plotting methods to extensive multiomic data through user-friendly commands and documented examples.

## INTRODUCTION

Public multiomic databases, such as The Cancer Genome Atlas (TCGA)^1^ and the cBioPortal repository,^2,3^ provide extensive data on the molecular landscape of cancer, but their incorporation in multiomic analyses has been hindered by the complexity of data coordination, selection, and management. The TCGA project generated multiomic data, including mutations, copy number variants, methylation, and gene expression quantification, for 33 human cancer types, while the cBioPortal public repository provides multiomic data for > 260 oncological studies in > 20 primary sites. The size and complexity of these databases impose time-consuming and technically complex barriers to the development of novel tools and analyses, even for advanced bioinformaticians. The lowering of these barriers requires new approaches to the distribution and management of large and complex data outputs.^4,5^

**CONTEXT**

**Key Objective**
To provide flexible, integrated, multiomic representations of public oncology databases in R/Bioconductor with greatly reduced data management overhead.
**Knowledge Generated**
Our Bioconductor software packages provide a novel approach to lower barriers to analysis and tool development for The Cancer Genome Atlas and cBioPortal databases.
**Relevance**
Our tools provide flexible, programmatic analysis of hundreds of fully integrated multiomic oncology data sets within an ecosystem of multiomic analysis tools.

**FIG 1.**
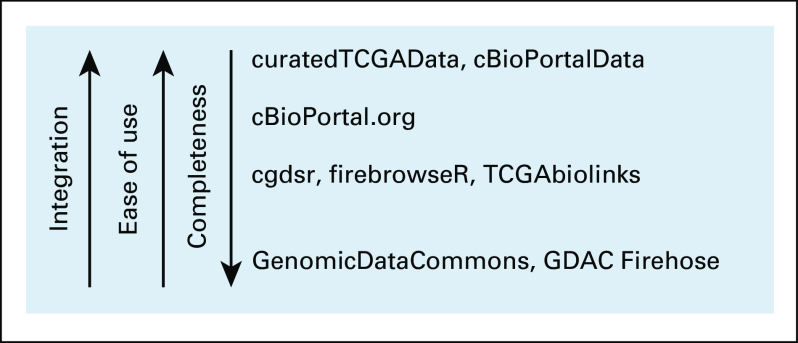
Comparison of The Cancer Genome Atlas (TCGA) data resources by integration, ease of use, and data completeness. Integration refers to the ability of the resource to be used within an analysis platform such as R and Bioconductor. A resource with high data completeness allows users to download the entirety of TCGA data. Ease of use is defined as the low cognitive overhead for use of a resource as imposed by data models and knowledge of query structures.

Existing command line resources such as the Genomic Data Commons (GDC),^6^ the Broad Institute’s GDAC Firehose pipeline tool, R packages such as firebrowseR,^7^ TCGAbiolinks,^8^ RTCGAToolbox,^9^ cgdsr,^10^ website interfaces such as cBioPortal,^2^ the Omics Discovery Index,^11^ and the GenomicDataCommons package^12^ provide varying degrees of portability, usability, and integration for multiomics data. However, in general, these resources either provide certain prespecified analyses but lack integration with platforms for statistical analysis or require significant effort to integrate the different data types within such a platform. They also present trade-offs between comprehensive data access and ease of use (Fig 1). Tools that provide comprehensive data access require familiarity with data models, linkage between sample and patient identifiers, and command line tools. Resources with high ease of use provide a more limited scope of data sets, and the responsibility to coordinate, manage, and even port multiple onco-omic data sets to analysis-ready platforms falls on the user.

We have implemented the curatedTCGAData, cBioPortalData, and TCGAutils packages to provide easily accessible multiomic data sets in the analysis-ready R^13^ and Bioconductor^14^ environment. The curatedTCGAData package serves integrated data sets for 33 different cancer types with > 11,000 tumor samples that are built on demand and contain selected data types as requested by the user. Where other platforms provide either comprehensive data acquisition or data subsets with limited analysis capabilities, curatedTCGAData provides a solid foundation for researchers looking to get started quickly with analyses of TCGA data across genomic assays and/or across different cancer types. cBioPortalData makes use of the cBioPortal web application programming interface (API) to serve integrative representations of multiomics data for > 260 and growing genomic studies. The TCGAutils package further provides facilities to make working with TCGA data easy with convenient identification, separation, and manipulation of sample and patient identifiers, leveraging the capabilities of the MultiAssayExperiment data structure.^15^

## METHODS

### Installation

The recommended installation procedure for Bioconductor packages is described in its installation instructions.^16^ These instructions detail the use of BiocManager, a Comprehensive R Archive Network package, for Bioconductor package installations. BiocManager allows easy installation of all three packages as follows: BiocManager::install(c(“curatedTCGAData”, “TCGAutils”, “cBioPortalData”).

Docker^17^ can be used to provide reproducible and easy-to-set up Bioconductor environments, using instructions provided from its download site.^18^ The Docker image provides an RStudio installation that can be used in conjunction with the aforementioned R package installation commands. Users are encouraged to run ‘BiocManager::valid()’ to verify that the Bioconductor installation and packages are up to date and properly installed.

### Data Structure Overview

Data sets from curatedTGCAData and cBioPortalData are represented using the established MultiAssayExperiment data structure^15^ that provides a framework for managing and organizing experimental assays on a set of samples in Bioconductor. The MultiAssayExperiment container eases the burden of data management by creating a graph representation of biological units and their relationship to multiple experiment measurements along with associated metadata. It provides a convenient platform from which to conduct integrative analyses while representing complex data structures and classes within the R and Bioconductor ecosystems.

Experiment data class representations are required to adhere to a set of minimal operations for compatibility. In particular, these data structures must be divisible by rows and columns and have discoverable dimension attributes, such as length and value labels. SummarizedExperiment is an example of a commonly supported Bioconductor class that is compatible with these basic requirements.^14^

The SummarizedExperiment class is the de facto standard representation for high-throughput genomic data in Bioconductor. It provides a flexible architecture that can support multiple experimental assays in a single instance. It also allows easy extensibility to other experimental data classes while maintaining the minimum requirements necessary for MultiAssayExperiment representation. One such extension of SummarizedExperiment is the RangedSummarizedExperiment structure. It supports structured genomic range representations as row metadata. MultiAssayExperiment supports an open-ended range of data classes despite class evolution.

### Preprocessing

**FIG A1.**
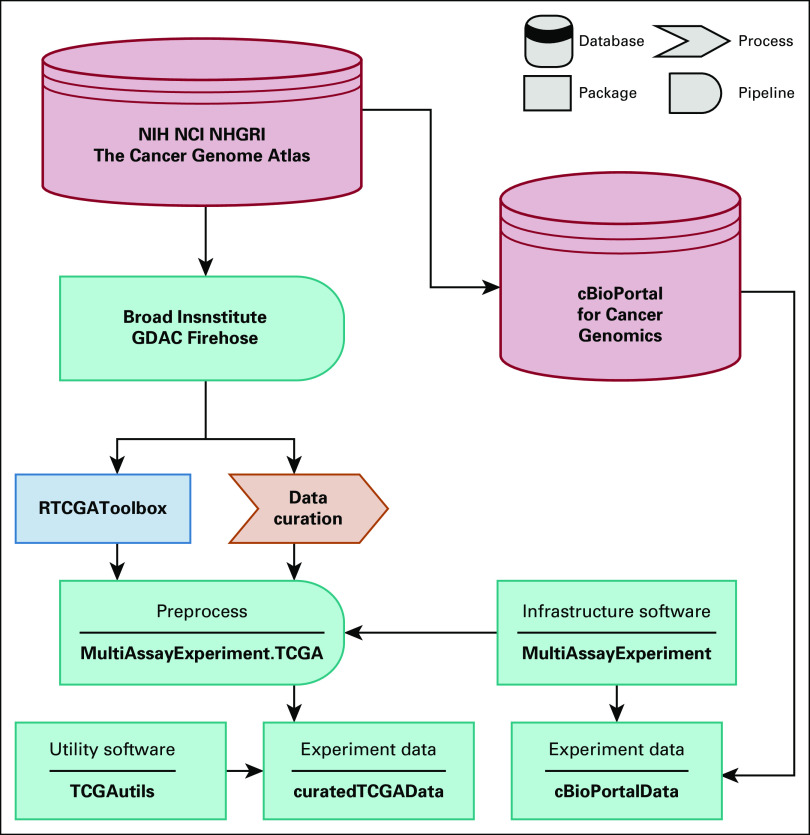
Flow diagram of the curatedTCGAData pipeline and cBioPortalData data provenance. NCI, National Cancer Institute; NHGRI, National Human Genome Research Institute; NIH, National Institutes of Health.

Data for approximately 11,000 samples and 33 different cancers were preprocessed, harmonized, and redistributed through curatedTCGAData. Data were first downloaded from the Broad Institute’s GDAC Firehose pipeline’s last run date (January 28, 2016)^19^ using the RTCGAToolbox Bioconductor package.^9^ Subtype information, taken from supplemental files of primary TCGA publications, was then added to the phenodata and uploaded to the cloud through Bioconductor’s ExperimentHub. Uploaded TCGA data were packaged into standard Bioconductor objects, such as SummarizedExperiment^20^ and RaggedExperiment^21^, classes that readily conform to MultiAssayExperiment container requirements. Appendix Figure A1 shows a schematic of the process from database to R/Bioconductor package.

The pipeline annotates ranged data with genome build information extracted from file names and annotation files where possible. It merges open-access tier, level 4, data with the more extensive merged level 1 clinical data, in some instances providing approximately 800 additional variables, while at the same time, removing columns where all values are missing and maintaining provenance of such column names in the metadata. Molecular subtype data were added to 19 of the 33 available cancer types (Appendix Table A1). Appendix Table A2 lists the available experimental assays and respective Bioconductor classes in curatedTCGAData. The open source curatedTCGAData pipeline is available through the MultiAssayExperiment download site.^22^ cBioPortalData serves data as provided by cBioPortal through its web API or through provided .gz files for complete data sets.

### ExperimentHub

The curatedTCGAData assembles data sets from components stored and served by ExperimentHub. After data extraction from RTCGAToolbox data representations and binning into appropriate Bioconductor data classes, the data were saved as serialized R data objects. Metadata were programmatically generated for each data type, and data for all 33 cancers were uploaded to the cloud using ExperimentHub, a Bioconductor-provided Amazon cloud storage service. The online Bioconductor data repository for experiment data is connected to and managed by an in-house database. This database is used by the ExperimentHub R package^23^ for the retrieval and download of queried data sets. ExperimentHub provides automatic local caching of the component R objects that are assembled by curatedTCGAData to create a MultiAssayExperiment, but these cached objects are not intended for direct use by the user.

**FIG A2.**
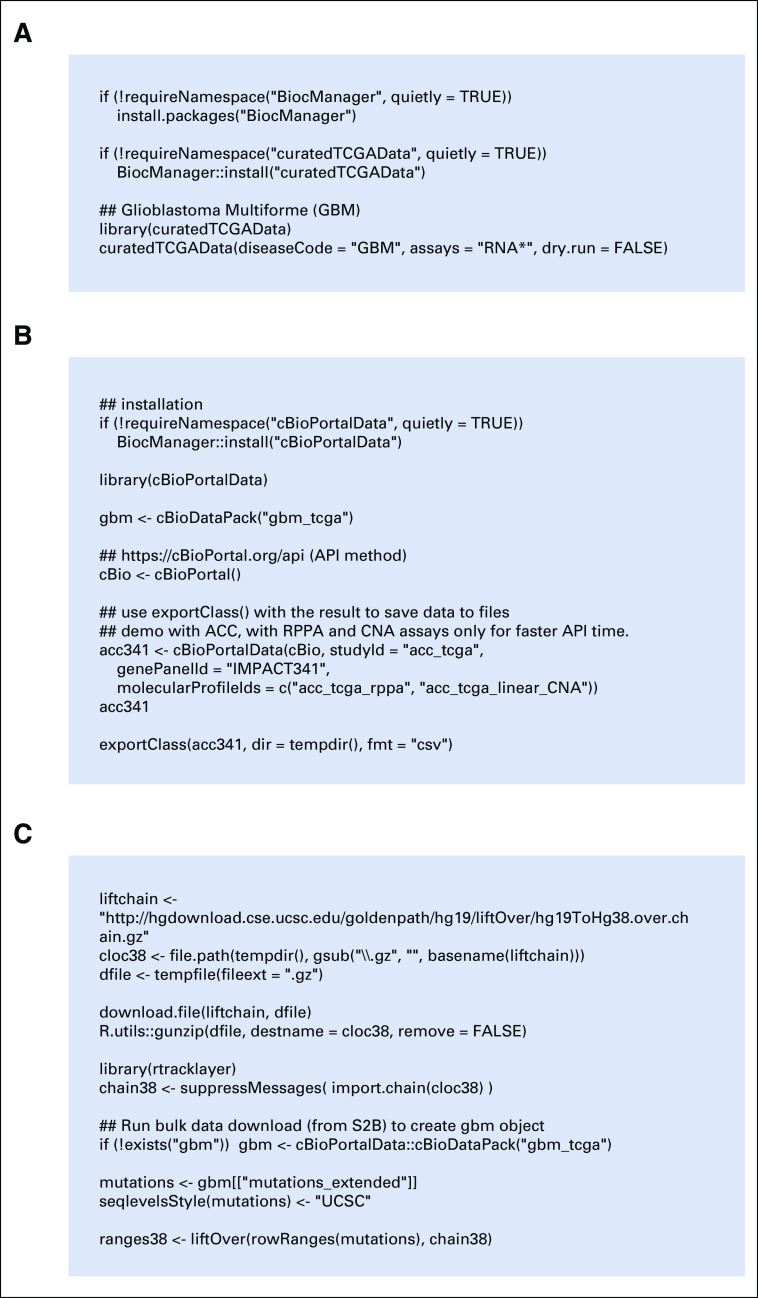
(A) Example code for installing and downloading The Cancer Genome Atlas (TCGA) data using curatedTCGAData. (B) Example cBioPortalData code for downloading and exporting TCGA data from cBioPortal and through the cBioPortal application programming interface (API). (C) Example hg19 to hg38 liftOver procedure using Bioconductor tools.

curatedTCGAData retrieves piecewise data representations and constructs a MultiAssayExperiment on the fly from ExperimentHub while ensuring that data across all requested experimental assays are accounted for and that imported data types conform to MultiAssayExperiment requirements through automatic class checks (Appendix Fig A2A). All data sets are harmonized to only include associated patient phenotype data for the requested assays.

### DelayedMatrix

To ensure efficient access, we used alternate data representations for methylation 450K and 27K assays because of their large size. curatedTCGAData makes use of the DelayedMatrix class from the DelayedArray package^24^ to represent such data. The hierarchical data format 5 (HDF5)–based^25^ DelayedMatrix representation avoids overconsumption of memory and allows users to load a “lazy” and partial representation of data on ordinary laptops. On ExperimentHub,^23^ methylation data sets are stored as two files: one provides the SummarizedExperiment shell, and the other contains the assay data in HDF5 through use of the saveHDF5SummarizedExperiment function in the SummarizedExperiment package.

### TCGAutils

**FIG A3.**
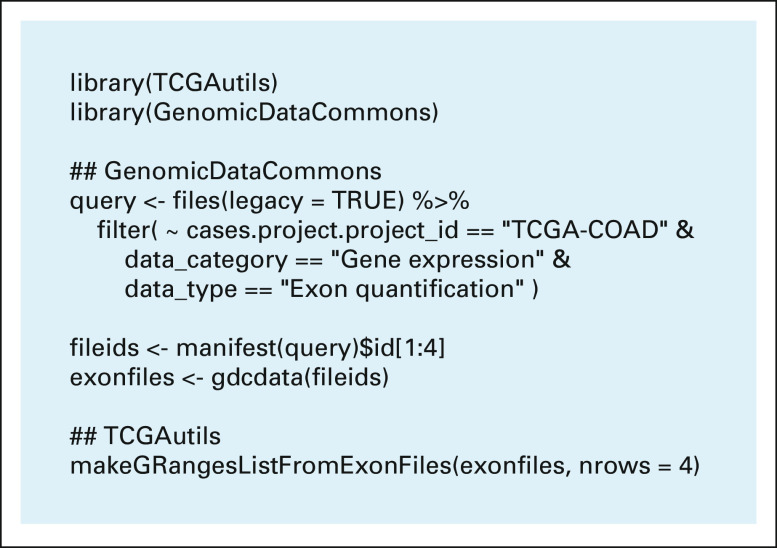
Example code for downloading data through GenomicDataCommons and loading with TCGAutils.

The TCGAutils package covers a wide variety of utility functions for simplified manipulation of TCGA data. This companion package is tailored to curatedTCGAData data sets but can also work with TCGA data sets, such as those obtained from cBioPortalData and the GDC (Appendix Figs A2B and A3). TCGAutils implements assay transformation functions that work on TCGA barcodes, such as splitAssays, to separate samples on the basis of type (eg, tumors, normals). We also provided annotation converter functions, such as mirToRanges, qreduceTCGA, and symbolsToRanges, for transforming microRNA metadata, summarizing mutation data, and converting gene symbols to genomic ranges, respectively. Several TCGA identifier functions, such as barcodeToUUID and TCGAbarcode, manipulate and translate TCGA barcodes to universal identifiers and vice versa.

### cBioPortalData

The cBioPortal for Cancer Genomics^26^ is an open access resource and open source platform for interactive and programmatic exploration of multiomic cancer data. The cBioPortal database currently provides > 260 data sets curated by the cBioPortal team, including TCGA and the International Cancer Genome Consortium.^3^ The cBioPortal API service^27^ provides programmatic access to the cBioPortal database, which is also used for in-house omics data management at several cancer centers, including the Memorial Sloan Kettering Cancer Center and the Dana-Farber Cancer Institute. The cBioPortalData package makes use of the cBioPortal API service to retrieve, cache, and subsequently integrate multiomic data as MultiAssayExperiment data objects. R/Bioconductor users do not need to construct API query operations to retrieve cBioPortal data; they only need to provide a study identifier and genes of interest to obtain a MultiAssayExperiment data set through the R interface. The cBioPortalData package can be installed as of Bioconductor release version 3.11.

### Differential Expression and Gene Set Enrichment Analysis

Upper quartile–normalized RNA-Seq by Expectation-Maximization transcripts per million gene expression values^28^ were obtained using curatedTCGAData. Analysis was restricted to 14 cancer types for which at least 10 adjacent normal tissue samples were available. While taking the pairing of samples (tumor *v* adjacent normal) into account, differential expression analysis was carried out on the basis of limma^29^ across the selected cancer types. Gene set enrichment analysis of Gene Ontology Biologic Process terms was performed using the over-representation test implemented in the EnrichmentBrowser package^30^ and contrasted with the results obtained from the application of Pathway Analysis with Down-weighting of Overlapping Genes (PADOG).^31^ Pan-cancer application of differential expression and gene set enrichment analysis was carried out using functionality from the GSEABenchmarkeR package.^32^

### Reproducible Research

All analyses presented in this article are reproducible using code provided online.^33^

## RESULTS

### Data and Software

The curatedTCGAData and cBioPortalData integrate data from two large public multiomic databases, using Bioconductor’s MultiAssayExperiment data structure^15^ (Appendix Fig A1). Multiassay and pan-cancer data sets are generated using a single R command that specifies the required data and returns a MultiAssayExperiment object (Appendix Fig A2A). curatedTCGAData accesses single-assay data sets processed from the GDAC Firehose pipeline and stored in Bioconductor’s ExperimentHub. The package integrates user-requested assays, cancer types, and clinicopathological data into a custom MultiAssayExperiment structure. cBioPortalData accesses data through two methods: through the cBioPortal web API, which enables downloading of a defined number of genes across a chosen number of oncological studies, and by parsing complete data sets downloaded as .zip files from cBioPortal. Both approaches use the MultiAssayExperiment representation to link multiomic profiles, enabling harmonized subsetting and flexible reshaping of data across assays and cancer types. This advance in integration improves flexibility and ease of use over other programmatic approaches to accessing these data (Fig 1).

TCGAutils provides an assortment of utility functions for working with MultiAssayExperiment data representations and TCGA-related data. The principal functionality allows users to convert genomic annotations to genomic ranges and positions, summarize genomic ranges of nonsilent mutations or copy number variations at the gene level, identify curated subtypes from primary TCGA publications, extract key level 4 clinical and pathological data from the hundreds or thousands of merged variables available, and produce OncoPrint plots. It also permits users to work with TCGA metadata by providing reference tables for TCGA barcodes and sample types, translating between TCGA patient and universal identifiers and separating selected specimens across assays. Other use cases in TCGAutils enable data imputation and text data conversion to standard Bioconductor data representations.

### Analysis Examples

**FIG 2.**
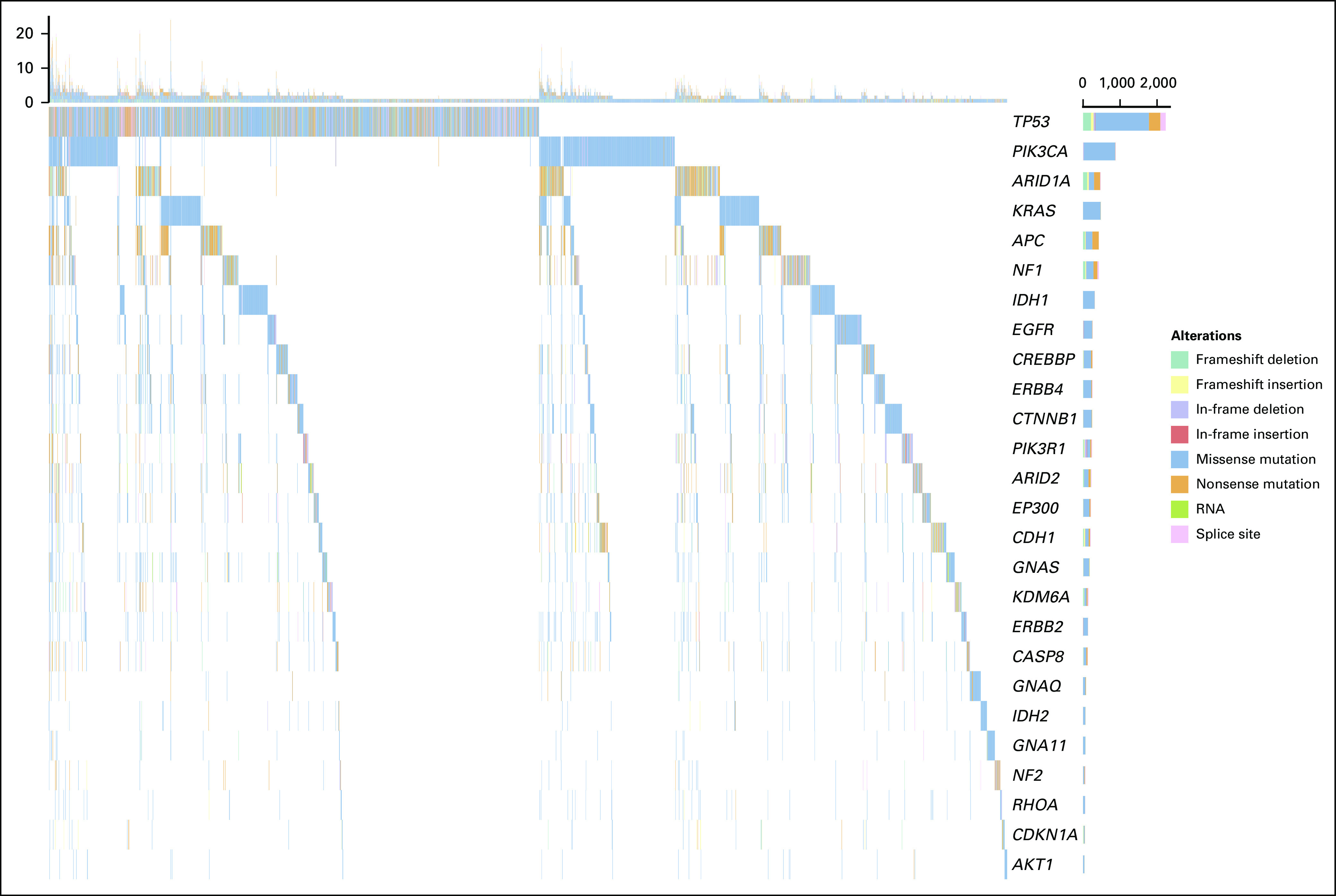
OncoPrint plot of selected cancer driver genes frequently mutated across 33 The Cancer Genome Atlas cancer types.

**FIG 3.**
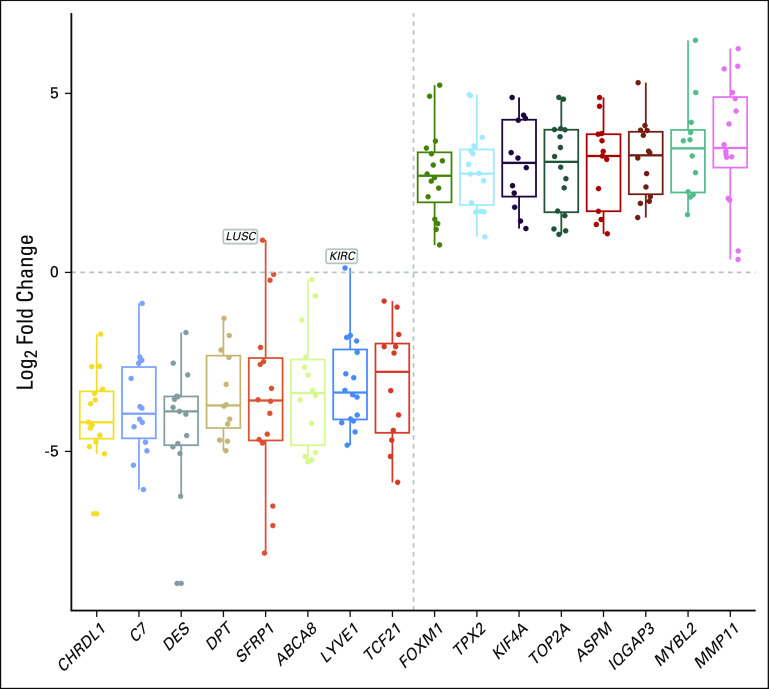
Pan-cancer differential expression analysis. Shown are the top eight consistently downregulated genes (bottom left) and the top eight consistently upregulated genes (top right) when comparing cancer versus adjacent normal samples across 14 cancer types.

**FIG 4.**
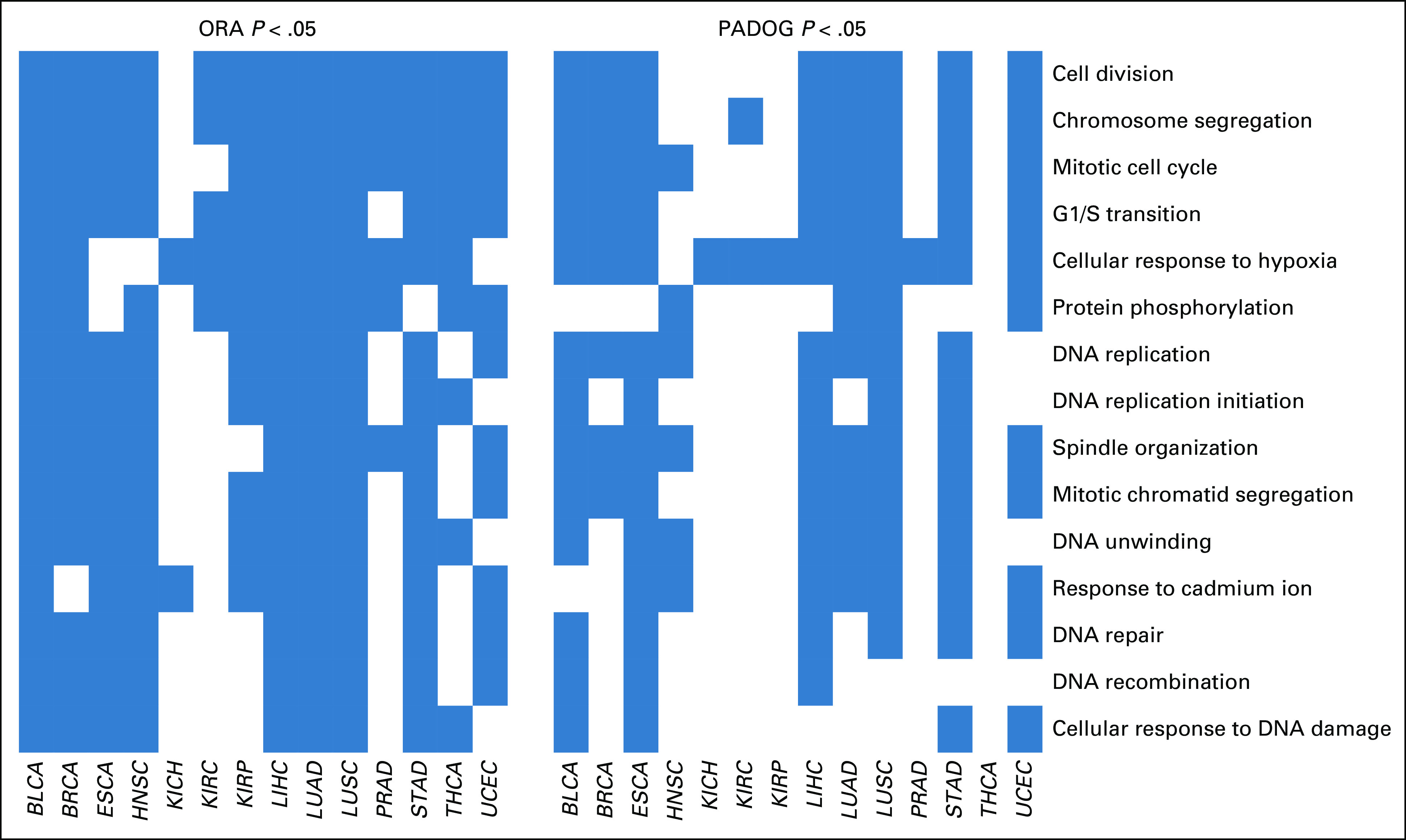
Pan-cancer gene set enrichment analysis. Shown are the 15 Gene Ontology Biologic Process terms that were most frequently found enriched for differential expression in cancer *v* adjacent-normal comparisons across 14 cancer types. On the left, enrichment is defined as being found by an over-representation analysis (ORA) with *P* < .05. For comparison, the right shows whether these terms were also found to be enriched according to another enrichment method (Pathway Analysis with Down-weighting of Overlapping Genes [PADOG]).

**FIG 5.**
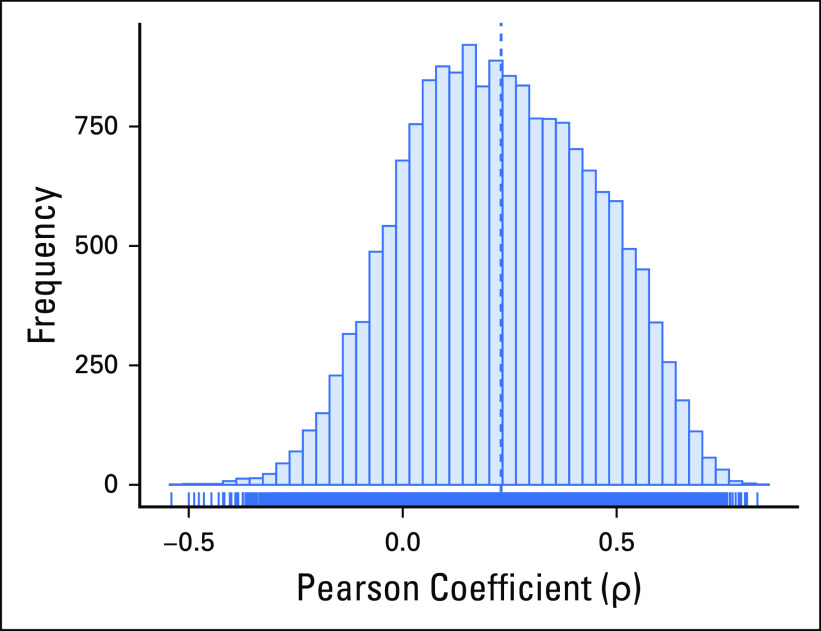
Histogram of the distribution of Pearson correlation coefficients between gene copy number and RNA sequencing gene expression in adrenocortical carcinoma. An integrative representation readily allows comparison and correlation of multiomics experiments.

**FIG 6.**
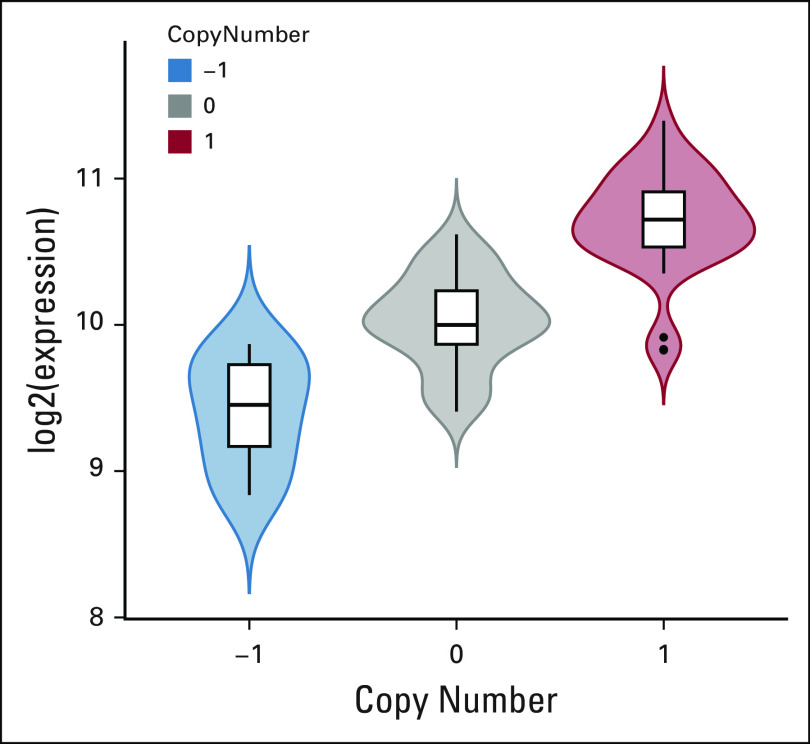
Gene dosage effect on *SNRPB2* expression in adrenocortical carcinoma (ACC) tumors. The violin plots show increasing expression of *SNRPB2* with increasing copy number, corresponding to a Pearson correlation of 0.83 (the highest correlation observed in ACC).

Several examples demonstrate the powerful and flexible analysis environment provided. These analyses, previously only achievable through a significant investment of time and bioinformatics training, become straightforward analysis exercises provided in an analysis vignette.^33^ First, we used curatedTCGAData to obtain the mutation data from all 33 cancers in TCGA, then isolated the 26 genes associated with tumor suppression and oncogenesis,^34^ and represented them by mutation type as an OncoPrint plot (Fig 2). This analysis is efficient and completely flexible, using the range-based representation of mutation data provided by curatedTCGAData. It confirms that *TP53* is the predominant gene, with mutations across many cancers and partially showing the mutual exclusivity of key driver mutations.^34,35^ Second, we performed a pan-cancer differential expression analysis across all TCGA cancer types against adjacent normal samples, showing the distribution of fold change across multiple cancer types for genes that are consistently up- and downregulated in cancer (Fig 3). This pan-cancer analysis can be performed in expressive steps of creating a MultiAssayExperiment containing all TCGA RNA sequencing (RNA-seq) data sets, filtering for primary tumors and adjacent normal tissues, and performing the differential expression analysis. We also performed a pan-cancer gene set enrichment analysis to identify Gene Ontology biological processes commonly activated or deactivated in multiple cancer types. We compared two common methods for enrichment analysis in Figure 4: over-representation analysis and PADOG. These analyses identify consistently altered molecular processes across multiple cancer types, including established hallmarks of cancer such as cell division and DNA repair.^36,37^ In an analysis involving multiple assay types, we calculated the bivariate correlation coefficients between gene copy number and RNA-seq expression values for adrenocortical carcinoma (Fig 5), observing a mostly positive distribution of correlations and showing that the expression of most genes is partially modulated by copy number. This analysis takes advantage of features to calculate the overlap between genomic ranges of copy number segments with genomic ranges of genes or any other genomic region. Finally, we showed the distribution of expression values by copy number for *SNRPB2*, the gene with the strongest relationship between expression and copy number in adrenocortical carcinoma (Fig 6).

## DISCUSSION

The availability of large-scale multiomics cancer data provides novel opportunities for integrative analysis. However, the integration, management, and statistical analysis of these resources remain challenging, even for advanced bioinformaticians. We present a set of data packages and software that makes multiomic analysis of TCGA data on 33 human cancers and cBioPortal data for > 260 onco-omic studies flexible, practical, and efficient for a broad range of bioinformatic, statistical, and epidemiological researchers. These data packages use established Bioconductor infrastructure, including SummarizedExperiment, MultiAssayExperiment, RaggedExperiment, and ExperimentHub, integrating multiomic data with clinicopathological data and simplifying analysis, visualization, and further tool development. curatedTCGAData and cBioPortalData link these data resources to an ecosystem of 26 Bioconductor packages for multiomic data analysis that require or suggest the MultiAssayExperiment data class. This ecosystem of packages, the companion package TCGAutils, and multiomic data management provided by MultiAssayExperiment simplify and extend the potential for novel multiomic analysis and tool development. The examples presented demonstrate significant simplification of previously expensive and challenging pan-cancer analyses, such as the identification of frequent mutations and recurrent differential gene expression across TCGA.

These resources serve a large amount of data, and several steps are made to make access and use more efficient. ExperimentHub provides automatic assay-level caching and avoids data redownload. TCGA methylation data files are stored in HDF5 out of memory; thus, users are able to load a MultiAssayExperiment with a small memory footprint of approximately 1 Gb for the most comprehensive cancer type in TCGA: breast invasive carcinoma. Users can also export the collected data within a MultiAssayExperiment object to text files through the exportClass function.

Because the GDAC Firehose pipeline primarily serves hg19 data, users who look to obtain hg38 build data are recommended to use tools such as the GDC,^6,12^ which can be integrated as MultiAssayExperiment objects with additional work. We also provide instructions to liftOver genomic coordinates from hg19 to hg38 using existing Bioconductor packages and associated chain files (Appendix Fig A2C and in the TCGAutils vignette). However, Gao et al^38^ compared legacy hg19-based (as procured by curatedTCGAData) and harmonized hg38-based (from the GDC) data sets in terms of biological interpretation and concluded that most analyses are largely insensitive to the update of genome build, with the most meaningful differences being in mutation calling algorithms and in mapping of methylation probes to noncoding genes.

## Figures and Tables

**FIG A4.**
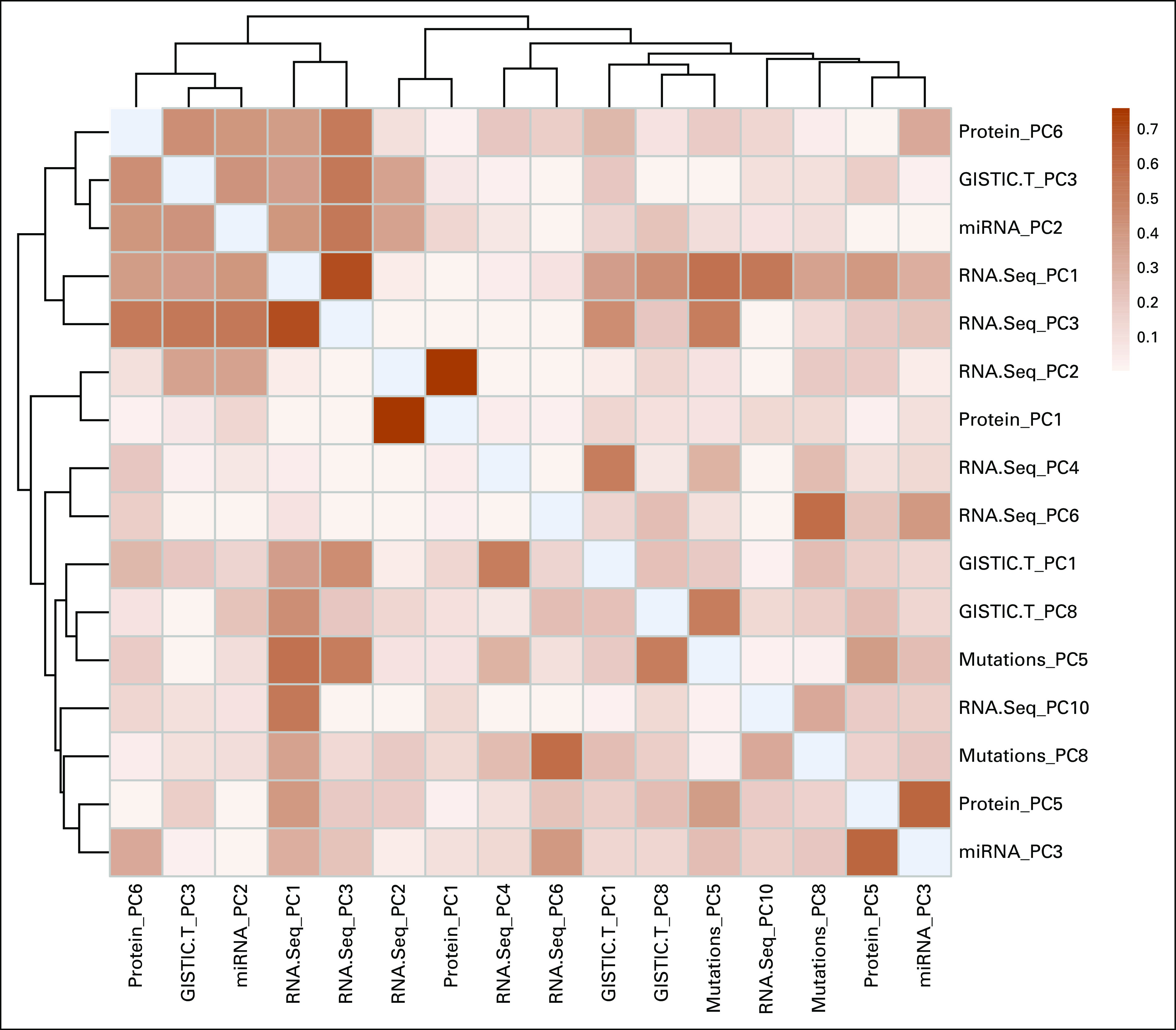
Correlated principal components (PCs) across experimental assays in adrenocortical carcinoma. miRNA, microRNA; RNA.Seq, RNA sequencing.

**TABLE A1.**
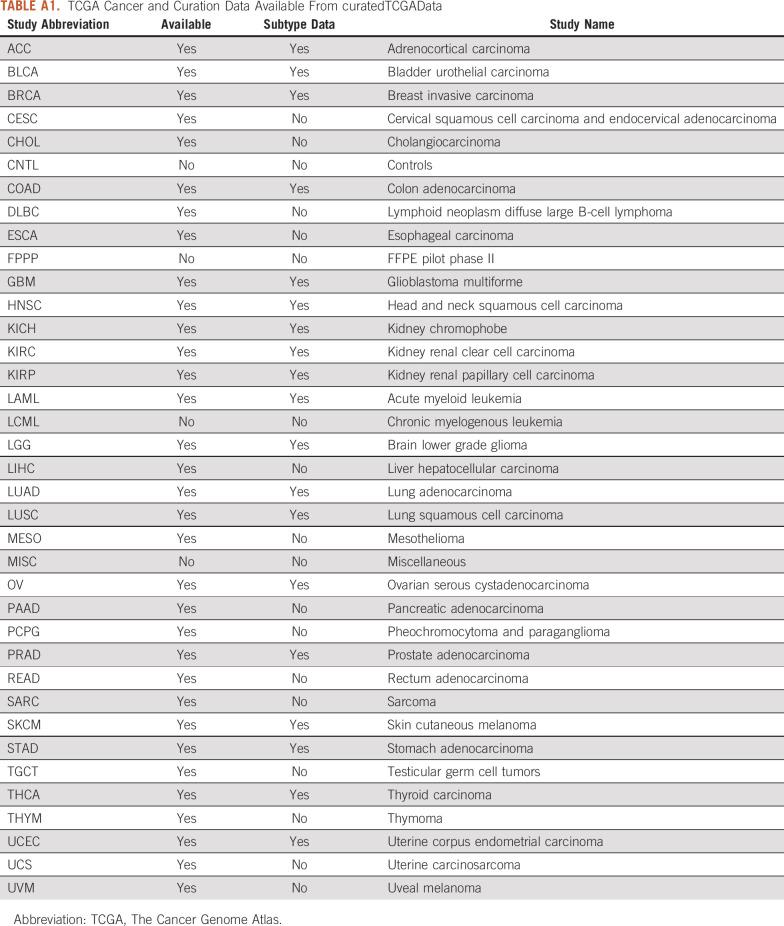
TCGA Cancer and Curation Data Available From curatedTCGAData

**TABLE A2.**
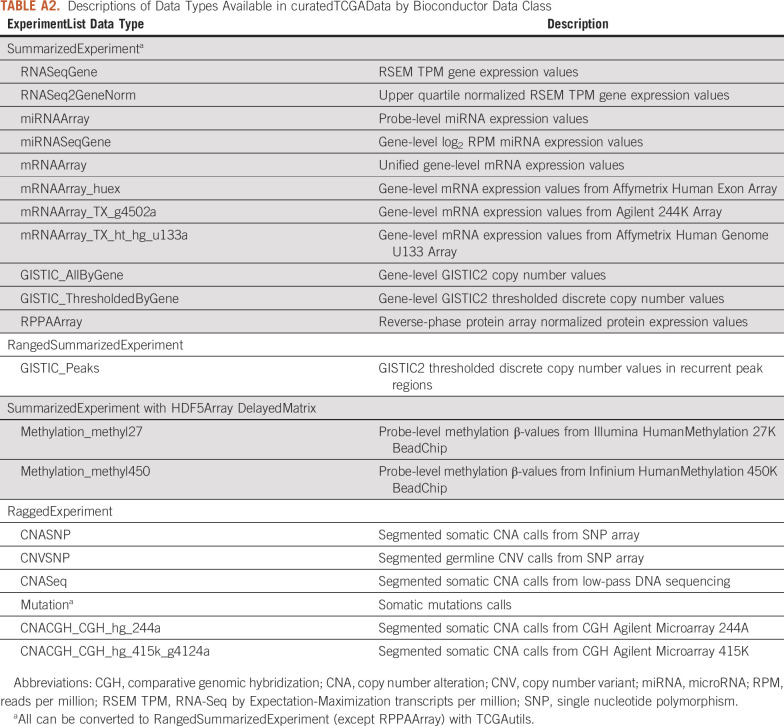
Descriptions of Data Types Available in curatedTCGAData by Bioconductor Data Class
